# Integrating primary care, shared decision making, and community engagement to facilitate equitable access to multi-cancer early detection clinical trials

**DOI:** 10.3389/fonc.2023.1307459

**Published:** 2024-02-29

**Authors:** Cheryl L. Thompson, Adam H. Buchanan, Ronald Myers, David S. Weinberg

**Affiliations:** ^1^ Penn State Cancer Institute, Department of Public Health Sciences, Pennsylvania State University College of Medicine, Hershey, PA, United States; ^2^ Department of Genomic Health, Geisinger, Danville, PA, United States; ^3^ Division of Population Science Department of Medical Oncology, Thomas Jefferson University, Philadelphia, PA, United States; ^4^ Department of Medicine, Fox Chase Cancer Center, Philadelphia, PA, United States

**Keywords:** multi-cancer detection assays, primary care, shared decision making, community engagement, cancer, screening, clinical trials

## Abstract

Effective implementation of cancer screening programs can reduce disease-specific incidence and mortality. Screening is currently recommended for breast, cervical, colorectal and lung cancer. However, initial and repeat adherence to screening tests in accordance with current guidelines is sub-optimal, with the lowest rates observed in historically underserved groups. If used in concert with recommended cancer screening tests, new biospecimen-based multi-cancer early detection (MCED) tests could help to identify more cancers that may be amendable to effective treatment. Clinical trials designed to assess the safety and efficacy of MCED tests to assess their potential for reducing cancer mortality are needed and many are underway. In the conduct of MCED test trials, it is crucial that participant recruitment efforts successfully engage participants from diverse populations experiencing cancer disparities. Strategic partnerships involving health systems, clinical practices, and communities can increase the reach of MCED trial recruitment efforts among populations experiencing disparities. This goal can be achieved by developing health system-based learning communities that build understanding of and trust in biomedical research; and by applying innovative methods for identifying eligible trial patients, educating potential participants about research trials, and engaging eligible individuals in shared decision making (SDM) about trial participation. This article describes how a developing consortium of health systems has used this approach to encourage the uptake of cancer screening in a wide range of populations and how such a strategy can facilitate the enrollment of persons from diverse patient and community populations in MCED trials.

## 1 Introduction

When effectively implemented, standard of care (SOC) cancer screening tests reduce disease-related morbidity and mortality (1–5). Unfortunately, observed reductions in mortality have not been distributed evenly across cancer types and diverse patient populations (6). Importantly, SOC cancer screening via periodic mammography, Pap testing, stool testing/colonoscopy, and low-dose CT scans is recommended for only four cancer types in the U.S. – breast, cervical, colorectal and lung. This leaves eight of the twelve most common cancer types without a recommended screening test. More than 60% of cancer deaths in 2022 are expected to have occurred for cancer types with no recommended screening test (7–9). Importantly, even for those cancers with a recommended screening test, adherence to screening is sub-optimal for all types, especially lung cancer (10). Furthermore, there are disparities in screening among medically underserved groups, with lower rates of screening and poorer outcomes in African American and Hispanic populations, as well as in populations residing in rural areas (6). Incomplete diagnostic follow-up of positive screening tests also limits the overall impact of SOC cancer screening (11).

Many populations with disparities in cancer screening also are underrepresented in clinical trials, including racial and ethnic minority groups, individuals of low socioeconomic status and rural residents (12, 13). Disparities are noted at eligibility, interest in participation and actual enrollment (14). There are many established reasons for lower enrollment of these groups, including transportation, cost, trust in medical system, and patient knowledge or comprehension of the research (15), and multi-level interventions are needed to increase enrollment of underrepresented populations (16).

Blood-based multi-cancer early detection (MCED) tests, alone or in concert with existing SOC screening modalities, have the potential to increase the detection of early, curable cancers and reduce disparities in screening. MCED tests, which analyze cell-free DNA and other biomarkers for the presence of cancer, have been shown in early trials to be capable of detecting cancers for which there is no SOC screening (17, 18). The collection of biospecimens, such as a simple blood test, that requires no preparation could improve adherence to cancer screening and, if distributed equitably, could reduce disparities in screening (11, 19). Further, MCED tests, by nature of being blood based, may be easier and cheaper to distribute across communities compared to large imaging equipment, such as mammography machines or CT scanners.

MCED tests are not currently part of routine cancer screening. They have not been approved by any of the regulatory bodies involved in evaluation of cancer screening tests. However, trials demonstrating clinical efficacy are underway around the world (20). Determining how to best implement broad use of these tests in diverse populations will require specific effort different than simply proving efficacy in selected populations. In this regard, initiatives like the National Cancer Institute’s Cancer Screening Research Network (CSRN) and The White House’s Cancer Moonshot programs are essential. It is crucial to develop new technology for screening and early detection in ways that do not increase, and preferably decrease, gaps in access and efficacy between populations. This involves ensuring that trials are conducted in diverse populations and settings with representation from communities that have traditionally been underrepresented in screening and other trials (21–24). For example, Jefferson Health (JH) has experience in using a theory-based model to guide the creation of a “health system learning community infrastructure” that can organize and adapt strategies for implementing interventions in primary care that can increase cancer screening and reduce disparities in screening use (25, 26). This learning community brings together health system leaders, healthcare providers, patients, insurance carriers, community stakeholders, and disease-specific advocacy groups. Evidence-based screening information and screening intervention implementation strategies and outcomes data are shared. Research project coordination (e.g., enrollment, longitudinal participant engagement and follow-up, data collection) is a key component of the learning community. The development of the CSRN for running large scale MCED trials presents an opportunity to establish this learning community on a broader scale. Here we describe examples of engaging with diverse community and clinician stakeholders in MCED trials and other cancer screening studies in hopes that these experiences will inform equitable MCED trial design.

## 2 Use of shared decision making in cancer screening and MCED trials

Shared decision making (SDM) and bidirectional communication between clinicians and potential research study participants can build trust in biomedical research. Mistrust in the health care system is disproportionally prevalent among racial and ethnic minority groups and members of underserved communities (27, 28). However, SDM can be challenging to implement well. The learning community model can be helpful for improving SDM for large scale trials with large numbers of providers involved in patient recruitment. SDM-based decision counseling has been used successfully in different health systems to advance colorectal cancer screening and prostate cancer screening research by guiding the recruitment of primary care practices and providers, delivery of decision counseling to patients, and engagement of diverse populations (29, 30). In a recent study on lung cancer screening, JH primary care physicians were encouraged to identify and refer patients who were eligible for lung cancer screening. Patients were randomized to one of three study groups: Outreach Contact plus Decision Counseling (OC-DC), Outreach Contact alone (OC), or usual care control group (UC). The research team found that screening was significantly higher in the combined OC/OC-DC group versus UC controls (5.5% vs. 1.8%, *p* = 0.001), where the UC group’s very low rates are similar to what is observed nationally (31). Screening was not statistically significantly higher in the OC-DC group than in the OC group (7% vs. 4%, respectively, *p* = 0.123). Screening referral and scheduling was also significantly higher in the OC/OC-DC group compared to controls (11% v. 5%, *p* = 0.001).

To examine the use of decision counseling related to MCED trial participation, a JH research team recently completed a prospective observational study with primary care patients at a large, urban healthcare system. Patients 50 to 80 years of age received mailed information about a planned MCED trial. After receiving this mailing, a research coordinator contacted patients by telephone, obtained consent, administered a survey, and completed a decision counseling interview to elicit factors affecting interest in the trial. The research coordinator also completed an endpoint telephone survey at one month to assess participant interest in and decisional conflict related to joining the trial. Of 1,000 eligible patients, we contacted 690 (69%). Of those contacted, 246 (36%) consented and completed the baseline survey; 217 (88%) completed decision counseling, and 219 completed the endpoint survey. On the endpoint survey, 177 (81%) respondents expressed interest in joining the MCED trial, and 162 (74%) reported low decisional conflict. Among 485 decision factors identified during decision counseling, 396 (82%) favored trial participation.

In concert with this effort, JH also completed a pilot study to assess primary care provider support for patient participation in a planned MCED test clinical trial. Information provided to primary care providers in an infographic explained that in a multi-year trial, health system research coordinators would be responsible for recruiting and following patients in the MCED trial. In addition, the infographic noted that study participants would undergo serial blood draws for MCED testing and would be randomly assigned to either a control group or an intervention group. The control group would receive usual care, with retrospective MCED test analysis; the intervention group would receive usual care, with immediate MCED test analysis and follow-up, as needed. Furthermore, it was explained that costs associated with testing and follow-up would be covered for both study groups. Providers who agreed to complete the survey (N=27) were asked if they intended to support patient participation in the MCED trial; 25 (93%) reported that they would support participation.

## 3 Use of community engagement in cancer screening and MCED trials

Community engagement is another important way to increase the reach of MCED trial participation, particularly for underrepresented individuals. Partnerships with community organizations and engagement with members and key stakeholders from underserved areas can help us identify eligible patients and communicate effectively with them, thereby diversifying representation in the trials. Earlier studies have shown that community-based participatory research can result in very high recruitment rates and very low refusal rates (32).

Over the past 20 years Fox Chase Cancer Center/Temple University has developed a research infrastructure linking over 400 community- and faith-based organizations with clinical partners across the PA-NJ region directed towards cancer screening within diverse Asian American populations (notably Chinese, Korean, and Vietnamese). Several community advisory boards inform collaboration and facilitate linkages among researchers, community members and clinical partners. These collaborations have resulted in successful completion of projects ranging from efforts intended to curb tobacco use, improve cervical cancer education or increase hepatitis B vaccination to broader questions like factors influencing clinical trials participation or willingness to contribute to biorepositories (33–37).

Numerous other examples of community engagement that improves cancer screening trial recruitment in underserved populations can be cited, including many studies aiming to account for cultural differences in a trial (38). For example, Wood et al. partnered with a members of a First Nations tribe to successfully enroll participants into a cervical cancer screening trial (39). These examples demonstrate that the positive impact of partnering closely with the communities from an early stage in the research development, empowering the community partners to help direct the research activities and doing so in a culturally sensitive manner.

Partnerships with federally qualified health centers (FQHCs) and other community hospitals represent another opportunity to enhance trial participation, particularly from underrepresented populations that are missed when recruitment is limited to academic medical centers. FQHCs serve patients regardless of their insurance status and ability to pay and can be an important source of primary and specialty care for many people, including 1/3 of people living in poverty (40).

## 4 Strategies for trial recruitment in rural communities in MCED trials

Members of rural communities are traditionally underrepresented in clinical trials as well. While this is influenced by much research happening in academic medical centers that are more commonly found in urban areas, other barriers to participation among rural residents, including concerns about access and knowledge of trials (41). In addition, researchers should consider ways of meeting community members where they are and bringing the trials to them instead of relying on under resourced individuals to come to academic medical centers.

Geisinger, which serves a largely rural area across 46 counties in central and northeastern Pennsylvania, has established a Multi-Cancer Screening Research Program to identify participants from the largely rural population it serves. An existing mobile team improves access to trials by traveling throughout the Geisinger service area and meeting potential participants in their communities. The team, which is responsible for conducting ongoing and upcoming MCED trials, includes a program manager, project managers responsible for recruitment, data collection, data transfer, and regulatory functions, as well as research assistants and phlebotomists. As demonstrated in the DETECT-A study (42), the Multi-Cancer Screening Research Program successfully enrolled 10,000 females in less than two years. The team’s approach included distributing study teams of research assistants and phlebotomists to 22 clinical and community sites across Geisinger’s geographic service area (43).

## 5 Discussion

Population-level cancer screening trials, specifically MCED trials, will require large consortia of multiple recruitment sites. Enrolling sufficient numbers of diverse participants is critical for their success. The strategies described above, including Learning Communities, community engagement, and efforts to improve access to trials in rural communities, represent promise for engaging diverse groups in large-scale MCED trials.

Community health workers (CHWs) represent another promising strategy that has not yet been applied to MCED trial recruitment. CHWs are trusted community members that understand the community that they live in and are advocates for better health in their community. CHWs represent a unique opportunity for researchers to engage with communities for trial enrollment as well. CHWs have repeatedly shown to enhance access to and quality of care in various setting, including primary care (44). However, few empirical trials have been done to test their effectiveness for increasing accrual to cancer screening trials. This represents a research opportunity specifically for MCED trials as well.

Although some trials of MCEDs are underway and others are planned, there are examples of successful studies that describe strategies for engaging primary care, facilitating SDM, and engaging communities that can increase the equitable representation of persons from diverse populations and underserved communities in cancer research. These studies and approaches suggest novel ways in which trial design and implementation strategies can be operationalized. Furthermore, using proven approaches in the context of health system learning communities could serve to amplify their effectiveness. Additional studies that explore these models and their use in addressing the recruitment of participants to MCED trails are needed. Using such evidence-based strategies will help us be more deliberate in our inclusion of individuals from underrepresented groups in trials, thereby making it possible to discern the effects of different MCED tests in diverse populations and set the stage for the equitable implementation of safe and effective tests in clinical practice.

## Data availability statement

The original contributions presented in the study are included in the article/supplementary material. Further inquiries can be directed to the corresponding author.

## Author contributions

CT: Conceptualization, Writing – original draft, Writing – review & editing. AB: Conceptualization, Writing – original draft, Writing – review & editing. RM: Conceptualization, Writing – original draft, Writing – review & editing. DW: Conceptualization, Writing – original draft, Writing – review & editing.

## References

[1] Zavala VA Bracci PM Carethers JM Carvajal-Carmona L Coggins NB Cruz-Correa MR. Cancer health disparities in racial/ethnic minorities in the United States. Br J Cancer (2021) 124(2):315–32. doi: 10.1038/s41416-020-01038-6 PMC7852513 32901135

[2] Aberle DR Adams AM Berg CD Black WC Clapp JD Fagerstrom RM. Reduced lung-cancer mortality with low-dose computed tomographic screening. N Engl J Med (2011) 365(5):395–409. doi: 10.1056/NEJMoa1102873 21714641 PMC4356534

[3] Vesco KK Whitlock EP Eder M Burda BU Senger CA Lutz K. Risk factors and other epidemiologic considerations for cervical cancer screening: a narrative review for the U.S. Preventive Services Task Force. Ann Intern Med (2011) 155(10):698–705, w216. doi: 10.7326/0003-4819-155-10-201111150-00377 22006929

[4] Lin JS Perdue LA Henrikson NB Bean SI Blasi PR. Screening for colorectal cancer: updated evidence report and systematic review for the US preventive services task force. Jama (2021) 325(19):1978–98. doi: 10.1001/jama.2021.4417 PMC13509038 34003220

[5] Lin JS Piper MA Perdue LA Rutter CM Webber EM O’Connor E. Screening for colorectal cancer: updated evidence report and systematic review for the US preventive services task force. Jama (2016) 315(23):2576–94. doi: 10.1001/jama.2016.3332 PMC13623242 27305422

[6] Nadauld L Goldman DP. Considerations in the implementation of multicancer early detection tests. Future Oncol (2022) 18(28):3119–24. doi: 10.2217/fon-2022-0120 36062430

[7] Clarke CA Patel AV Kurian AW Hubbell E Gomez SL. Racial/Ethnic Differences in Cancer Diagnosed after Metastasis: Absolute Burden and Deaths Potentially Avoidable through Earlier Detection. Cancer Epidemiol Biomarkers Prev (2022) 31(3):521–7. doi: 10.1158/1055-9965.Epi-21-0823 PMC9381115 34810206

[8] Singh GK Williams SD Siahpush M Mulhollen A. Socioeconomic, rural-urban, and racial inequalities in US cancer mortality: part I-all cancers and lung cancer and part II-colorectal, prostate, breast, and cervical cancers. J Cancer Epidemiol (2011) 2011:107497. doi: 10.1155/2011/107497 22496688 PMC3307012

[9] Siegel RL Miller KD Fuchs HE Jemal A. Cancer statistics, 2022. CA Cancer J Clin (2022) 72(1):7–33. doi: 10.3322/caac.21708 35020204

[10] Rai A Doria-Rose VP Silvestri GA Yabroff KR. Evaluating lung cancer screening uptake, outcomes, and costs in the United States: challenges with existing data and recommendations for improvement. J Natl Cancer Inst (2019) 111(4):342–9. doi: 10.1093/jnci/djy228 PMC6657438 30698792

[11] Loomans-Kropp HA Umar A. Cancer prevention and screening: the next step in the era of precision medicine. NPJ Precis Oncol (2019) 3:3. doi: 10.1038/s41698-018-0075-9 30701196 PMC6349901

[12] Murthy VH Krumholz HM Gross CP. Participation in cancer clinical trials: race-, sex-, and age-based disparities. JAMA (2004) 291(22):2720–6. doi: 10.1001/jama.291.22.2720 15187053

[13] Baquet CR Commiskey P Daniel Mullins C Mishra SI. Recruitment and participation in clinical trials: socio-demographic, rural/urban, and health care access predictors. Cancer Detect Prev (2006) 30(1):24–33. doi: 10.1016/j.cdp.2005.12.001 16495020 PMC3276312

[14] Caston NE Lalor F Wall J Sussell J Patel S Williams CP. Ineligible, unaware, or uninterested? Associations between underrepresented patient populations and retention in the pathway to cancer clinical trial enrollment. JCO Oncol Pract (2022) 18(11):e1854–e65. doi: 10.1200/OP.22.00359 PMC9653198 36178922

[15] McPhee NJ Nightingale CE Harris SJ Segelov E Ristevski E. Barriers and enablers to cancer clinical trial participation and initiatives to improve opportunities for rural cancer patients: A scoping review. Clin Trials (2022) 19(4):464–76. doi: 10.1177/17407745221090733 35586873

[16] Hamel LM Penner LA Albrecht TL Heath E Gwede CK Eggly S. Barriers to clinical trial enrollment in racial and ethnic minority patients with cancer. Cancer Control (2016) 23(4):327–37. doi: 10.1177/107327481602300404 PMC5131730 27842322

[17] Lennon AM Buchanan AH Rego SP Choudhry OA Elias PZ Sadler JR. Outcomes in participants with a false positive multi-cancer early detection (MCED) test: Results from >4 years follow-up from DETECT-A, the first large, prospective, interventional MCED study. J Clin Oncol (2023) 41(16_suppl):3039. doi: 10.1200/JCO.2023.41.16_suppl.3039

[18] Schrag D Beer TM McDonnell CH 3rd Nadauld L Dilaveri CA Reid R. Blood-based tests for multicancer early detection (PATHFINDER): a prospective cohort study. Lancet (2023) 402(10409):1251–60. doi: 10.1016/S0140-6736(23)01700-2 PMC11027492 37805216

[19] Davis KV Hallman MH DiCarlo M Wambua SM Jaffe RL Welsh AW. Factors likely to affect the uptake of genomic approaches to cancer screening in primary care: A scoping review. J Pers Med (2022) 12(12):2044. doi: 10.3390/jpm12122044 36556264 PMC9785136

[20] Nicholson BD Oke J Virdee PS Harris DA O’Doherty C Park JE. Multi-cancer early detection test in symptomatic patients referred for cancer investigation in England and Wales (SYMPLIFY): a large-scale, observational cohort study. Lancet Oncol (2023) 24(7):733–43. doi: 10.1016/S1470-2045(23)00277-2 37352875

[21] Duma N Vera Aguilera J Paludo J Haddox CL Gonzalez Velez M Wang Y. Representation of minorities and women in oncology clinical trials: review of the past 14 years. J Oncol Pract (2018) 14(1):e1–e10. doi: 10.1200/JOP.2017.025288 29099678

[22] Abdul Latip SNB Chen SE Im YR Zielinska AP Pawa N. Systematic review of randomised controlled trials on interventions aimed at promoting colorectal cancer screening amongst ethnic minorities. Ethn Health (2023) 28(5):661–95. doi: 10.1080/13557858.2022.2139815 36352539

[23] Kunitomo Y Bade B Gunderson CG Akgun KM Brackett A Tanoue L. Evidence of racial disparities in the lung cancer screening process: a systematic review and meta-analysis. J Gen Intern Med (2022) 37(14):3731–8. doi: 10.1007/s11606-022-07613-2 PMC9585128 35838866

[24] Sokale IO Montealegre JR Oluyomi AO Thrift AP. Trends and racial/ethnic differences in predictors of cervical cancer screening among US women ages 30-64 years. Cancer Epidemiol Biomarkers Prev (2023) 32(1):82–90. doi: 10.1158/1055-9965.EPI-22-0970 36306382 PMC9839647

[25] Myers RE DiCarlo M Romney M Fleisher L Sifri R Soleiman J. Using a health system learning community strategy to address cancer disparities. Learn Health Syst (2018) 2(4):e10067. doi: 10.1002/lrh2.10067 31245591 PMC6508848

[26] Man LC DiCarlo M Lambert E Sifri R Romney M Fleisher L. A learning community approach to identifying interventions in health systems to reduce colorectal cancer screening disparities. Prev Med Rep (2018) 12:227–32. doi: 10.1016/j.pmedr.2018.10.009 PMC6202664 30370210

[27] Boulware LE Cooper LA Ratner LE LaVeist TA Powe NR. Race and trust in the health care system. Public Health Rep (2003) 118(4):358–65. doi: 10.1093/phr/118.4.358 PMC1497554 12815085

[28] Musa D Schulz R Harris R Silverman M Thomas SB. Trust in the health care system and the use of preventive health services by older black and white adults. Am J Public Health (2009) 99(7):1293–9. doi: 10.2105/AJPH.2007.123927 PMC2696665 18923129

[29] Myers RE Daskalakis C Kunkel EJ Cocroft JR Riggio JM Capkin M. Mediated decision support in prostate cancer screening: a randomized controlled trial of decision counseling. Patient Educ Couns (2011) 83(2):240–6. doi: 10.1016/j.pec.2010.06.011 20619576

[30] Myers RE Stello B Daskalakis C Sifri R Gonzalez ET DiCarlo M. Decision support and navigation to increase colorectal cancer screening among hispanic patients. Cancer Epidemiol Biomarkers Prev (2019) 28(2):384–91. doi: 10.1158/1055-9965.EPI-18-0260 30333221

[31] Pham D Bhandari S Oechsli M Pinkston CM Kloecker GH. Lung cancer screening rates: Data from the lung cancer screening registry. J Clin Oncol (2018) 36(15_suppl):6504. doi: 10.1200/JCO.2018.36.15_suppl.6504

[32] Greiner KA Friedman DB Adams SA Gwede CK Cupertino P Engelman KK. Effective recruitment strategies and community-based participatory research: community networks program centers’ recruitment in cancer prevention studies. Cancer Epidemiol Biomarkers Prev (2014) 23(3):416–23. doi: 10.1158/1055-9965.EPI-13-0760 PMC3971731 24609851

[33] Ma GX Shive SE Tan Y Toubbeh JI Fang CY Edwards RL. Tobacco use, secondhand smoke exposure and their related knowledge, attitudes and behaviors among Asian Americans. Addict Behav (2005) 30(4):725–40. doi: 10.1016/j.addbeh.2004.08.018 15833577

[34] Fang CY Lee M Feng Z Tan Y Levine F Nguyen C. Community-based cervical cancer education: changes in knowledge and beliefs among Vietnamese American women. J Community Health (2019) 44(3):525–33. doi: 10.1007/s10900-019-00645-6 PMC6529234 30915676

[35] Gao W Ma GX Tan Y Fang C Weaver J Jin M. Factors associated with willingness to participate in biospecimen research among Chinese Americans. Biopreserv Biobank (2014) 12(2):131–8. doi: 10.1089/bio.2013.0081 PMC3995351 24749880

[36] Ma GX Fang CY Shive SE Toubbeh J Tan Y Siu P. Risk perceptions and barriers to Hepatitis B screening and vaccination among Vietnamese immigrants. J Immigr Minor Health (2007) 9(3):213–20. doi: 10.1007/s10903-006-9028-4 17265128

[37] Ma GX Seals B Tan Y Wang SY Lee R Fang CY. Increasing Asian American participation in clinical trials by addressing community concerns. Clin Trials (2014) 11(3):328–35. doi: 10.1177/1740774514522561 PMC4156927 24603005

[38] Mitchell SM Pedersen HN Sekikubo M Biryabarema C Byamugisha JJ Mwesigwa D. Strategies for community education prior to clinical trial recruitment for a cervical cancer screening intervention in Uganda. Front Oncol (2016) 6:90. doi: 10.3389/fonc.2016.00090 27148482 PMC4829601

[39] Wood B Burchell AN Escott N Little J Maar M Ogilvie G. Using community engagement to inform and implement a community-randomized controlled trial in the anishinaabek cervical cancer screening study. Front Oncol (2014) 4:27. doi: 10.3389/fonc.2014.00027 24600584 PMC3928568

[40] HRSA. Health Center Program: Impact and Growth (2023). Available at: https://bphc.hrsa.gov/about-health-centers/health-center-program-impact-growth.

[41] Kim SH Tanner A Friedman DB Foster C Bergeron CD. Barriers to clinical trial participation: a comparison of rural and urban communities in South Carolina. J Community Health (2014) 39(3):562–71. doi: 10.1007/s10900-013-9798-2 24310703

[42] Lennon AM Buchanan AH Kinde I Warren A Honushefsky A Cohain AT. Feasibility of blood testing combined with PET-CT to screen for cancer and guide intervention. Science (2020) 369(6499):eabb9601. doi: 10.1126/science.abb9601 32345712 PMC7509949

[43] Honushefsky A WE Sheridan K Spickard KM LeMasters MR Walter CN Beaver T. Real-time evaluation and adaptation to facilitate rapid recruitment in a large cohort 2023. doi: 10.1101/2023.01.30.23285102v1 PMC10938733 38481315

[44] Hartzler AL Tuzzio L Hsu C Wagner EH. Roles and functions of community health workers in primary care. Ann Fam Med (2018) 16(3):240–5. doi: 10.1370/afm.2208 PMC5951253 29760028

